# Experiences of pregnancy in adolescence of internally displaced women in Bogotá: an ethnographic approach

**DOI:** 10.1186/s12978-020-0889-0

**Published:** 2020-03-02

**Authors:** Yazmin Cadena-Camargo, Anja Krumeich, Maria Claudia Duque-Páramo, Klasien Horstman

**Affiliations:** 1 Research school CAPHRI, Department of Health, Ethics, and Society, Faculty of Health, Medicine, and Life Sciences, Maastricht University, Maastricht, The Netherlands; 2 Faculty of Medicine, Department of Preventive and Social Medicine, Pontificia Universidad Javeriana, Bogotá, Colombia; 3 Bogotá, Colombia

**Keywords:** Adolescent pregnancy, Teenage pregnancies, Internally displaced persons (IDPs)

## Abstract

**Background:**

Pregnancy in adolescence is higher among internally displaced women in Colombia than non-displaced women. It is defined as a problem with significant negative outcomes by both biomedical and epidemiological approaches. However, little is known about pregnancy during adolescence from the perspective of women who experienced this in the specific context of armed conflict and displacement.

**Aim:**

This article focuses on how internally displaced women understand their experiences of pregnancy in adolescence in the context of armed conflict through an ethnographic approach in a receptor community of internally displaced women in Bogotá, Colombia.

**Methods:**

Based on 10 years of experience in the community, we conducted 1 year of fieldwork, using an ethnographic approach. We collected life stories of 20 internally displaced women through in-depth interviews and ran 8 workshops with them and other women from the community. We used thematic analysis to analyse the responses of internally-displaced women and understand how they made meaning around their experiences of adolescent pregnancy in the context of displacement.

**Results:**

The main themes that emerged from participants’ experiences include rural violence, early family life (characterized by violence and mistreatment at home), meanings of pregnancy at an early age (including being challenged and feelings of love), and reactions to their pregnancies during adolescence (such as stigmatization) from their families and partners.

**Conclusion:**

Our analysis of the in-depth interviews and the workshops suggests that adolescent pregnancy among women who are internally displaced has complex dynamics, characterized by the violent context of the rural areas, but primarily by the violence experienced during their childhood. The experience of pregnancy during adolescence brings feelings of ownership and also challenges, together with the forced displacement. This understanding will provide insights for policy makers and healthcare providers on how to work with this specific population who have experienced pregnancy in adolescence.

## Resumen

**Antecedentes:**

El embarazo en la adolescencia es mayor en mujeres en condición de desplazamiento interno en Colombia, con respecto a las no desplazadas. Dicha condición es definida como un problema con consecuencias significativas desde el punto de vista biomédico y epidemiológico. Sin embargo, es necesario indagar estas experiencias desde las perspectivas de las mujeres que lo han experimentado, específicamente en el contexto del conflicto armado y del desplazamiento.

**Objetivo:**

Este artículo tiene por objeto comprender cómo interpretan las mujeres en condición de desplazamiento interno sus propias experiencias de embarazo en la adolescencia en el contexto del conflicto armado Colombiano a través de una aproximación etnográfica en una comunidad receptora en Bogotá.

**Métodos:**

Con base en 10 años de experencia trabajando en la comunidad, realizamos un año de trabajo de campo a través de observación participante, 20 entrevistas a profundidad para reconstruir sus historias de vida y 8 talleres con las mujeres en condición de desplazamiento en un barrio en Ciudad Bolivar, en Bogotá. Realizamos análisis temático de la información para analizar los datos de las mujeres desplazadas y explorar como ellas entendieron sus experiencias de embarazo en la adolescencia tras el contexto del desplazamiento.

**Resultados:**

Los principales temas que emergieron de las experiencias de las participantes incluyeron la violencia en áreas rurales, su niñez (incluso los significados de embarazo a temprana edad y el enamoramiento), y las diferentes reacciones frente al embarazo (como la estigmatización) por parte de sus familias y sus parejas.

**Conclusión:**

El embarazo en la adolescencia en mujeres en condición de desplazamiento interno tiene dinamicas complejas e incluye el contexto violento que viven en las areas rurales de donde son originarias, pero principalmente la violencia que vivieron durante su niñez. La experiencia de embarazo durante la adolescencia les genera sentimientos de pertenencia y las anima a sobrellevar las condiciones del desplazamiento. Estos hallazgos pueden ayudar en la construcción de políticas públicas y al personal de salud para trabajar con esta población.

## Plain English summary

Pregnancy during adolescence among internally displaced women is a big concern with medical social and economic consequences. This study provides an understanding of this situation from the point of view of internally displaced women who were pregnant as adolescents in Bogotá. We found in our analysis of the narratives, interviews, and workshops that the context of violence and armed conflict in the rural areas and the violence experienced during childhood represent the main characteristics involved on their pregnancies. Nevertheless, participants’ experience of early pregnancies encourages them to continue overcoming all the situations they live through as displaced women.

## Introduction

According to the national health survey in Colombia, the proportion of adolescents who were pregnant was higher among internally displaced persons (IDPs) than among adolescent population who has not been forced to migrate [1]. In Colombia, 13.8% of internally displaced girls aged 15–16 are pregnant, as compared to 7.5% of girls of the same age who have not been displaced. Among girls aged 17–19, 36.4% of internally displaced girls have experienced pregnancy, compared to 28.4% of non-internally displaced girls of the same age [1–4]. This occurs in a country where the overall incidence of pregnancy in adolescence is still high, with a prevalence of 19,5% – one of the highest in Latin America [2–4].

In 2017, Villarán introduced the notion of ‘possible pathways of pregnancies’ [5] to explain how girls understand early pregnancies from their point of view in Lima, Perú. She includes in her analysis the affective dependence of the girls to their partners, linked with the absence of girls’ parents, and forced relationships and rapes [5]. Despite the studies on pregnancies during adolescence, there is a lack of information about this topic in this specific population of internally displaced women in Bogotá, and how they understand it based on their experiences and from their point of view.

We will start by sketching out the different approaches to understanding pregnancy in adolescence, including biomedical and epidemiological ones, and different currents of thought within the anthropological approach. Next, we will explain the methodology and the ethical framing of the study. After presenting the results, we will discuss them in relation to current discourses on adolescent pregnancies.

### Approaches towards pregnancy in adolescence

In understanding pregnancy in adolescence, biomedical and epidemiological perspectives have thus far been dominant. In biomedical literature, pregnancy in adolescence is primarily defined as a problem with negative consequences for maternal, perinatal, and infant mortality [6–8]. Some authors have described the association between pregnancy in adolescence and medical complications and undesirable medical outcomes [9–11]. In a similar vein, other authors discuss how pregnancy in adolescence relates to negative individual consequences, such as fewer/lesser economic opportunities, family and social rejection, emotional difficulties and educational underachievement [12, 13]. In addition, the World Health Organization describes how pregnancy in adolescence contributes to the vicious cycle of ill-health and poverty [7]. In epidemiological studies, pregnancy in adolescence is primarily framed as a problem to prevent. These perspectives often zoom in on risk factors for pregnancy in adolescence such as low educational level, lack of information about sexual health and family planning, cultural perceptions about motherhood, low socioeconomic status, a non-nuclear family structure, violence at home, lack of parental supervision, early initiation of sexual intercourse, and cultural and regional factors [4, 14, 15].

While these approaches focus on the social impacts of adolescent pregnancy, we wonder how adolescents themselves understand “the possible pathways of pregnancies” and how they experience early motherhood. Regarding ‘the possible pathways of pregnancies’, the majority of studies have pictured the adolescents as victims responsible for what happened to them. For example, Ivey found that females who experience social isolation, unrealistic expectations regarding home responsibilities, lack of control and limited opportunity for decision-making or plans for the future may be at risk for pregnancy in adolescence [16]. In addition, Mora Cancino, in a study conducted in Mexico argued that pregnancy in adolescence was associated with ‘other antisocial behaviours’, such us alcohol and drugs consumption, as well as criminal actions, showing their low level of acceptation for the social norms in their society [17]. Regarding experiences of motherhood, some studies also convey negative perceptions of adolescents. That is the case with one study in Uganda, which showed the difficulties for adolescents in coping with the stress of the transition from adolescence to parenthood. The adolescents also expressed regrets that pregnancy had reduced their opportunities in life, and regretted the decision to get into relationships, conceive or go into motherhood at an early age [18]. On the other hand, some authors have different and more positive findings, ascribing agency and the “pathway of pregnancies” as a positive choice and care. In the United Kingdom, Anwar found that the young age of girls did not affect their ability to be competent mothers. Rather than being the outcome of low expectations, these young women could see the social and personal fulfilment of having a baby [19]. In a study of African and Australian teenage mothers, motherhood brought happiness for many of the young women, despite the associated challenges of early parenting, along with an associated sense of maturity and responsibility [20].

Furthermore, according to Anastas [21], different perspectives on pregnancy in adolescence can be classified with Kelly’s typology. Kelly distinguishes three approaches of adolescent pregnancy. Type A includes all the articles where teenage pregnancies can be summarized as the expression: “Something is wrong with the girl.” This group of studies includes lack of knowledge among girls about how to prevent an unwanted pregnancy, immature expectations of what being a mother is going to be like, past or present trauma including sexual abuse and other psychological problems. Type B is resumed as “The wrong kind of family,” where the girl’s family of origin or her subculture failed to convey “good” values about sexuality and preferred family forms. These studies remain with the concern over the fate of the nuclear family. And Type C describes as “dissenting views” which is advanced from feminist, Afrocentric, and other critical standpoints, where becoming a mother can be a rational route to attaining adult status and independence [21].

In Colombia, there are some anthropological studies regarding pregnancy in adolescence. De la Cuesta argued that pregnancy in adolescence in Colombia occurs in the context of a relationship where rules of romantic love and gender guide the behaviour of girls and men [22]. The author explains that sexual relationships are related to their identity as women, and the transition from girls to mature women. The role of men is to introduce girls into romantic and sexual love [22]. In addition, Pacheco concludes that pregnancy in adolescence occurs because of a lack of knowledge of family planning methods, romantic love ideals, and willingness to have a family that guides them towards having different partners and children [23]. The same author in another study explains how pregnancy is seen by society as a mistake where the girl is guilty and feels condemnation [24]. Similarly, Quintero explains how girls who become pregnant do not feel prepared for the big responsibilities, and also that they cannot do the things they did prior to pregnancy, such as going out to parties [25]. These studies are related to the A and B of Kelly’s typology, and contain more negative than positive understandings about early pregnancy.

While epidemiological studies show a rise of pregnancy in adolescence among internally displaced girls, anthropological studies in Colombia have not yet addressed this specific context. The number of displaced persons in Colombia who are affected by internal migration is one of the highest in the world [26, 27] leading to 6.7 million long-term internally displaced persons (IDPs), from which approximately 300,000 are living in Bogotá [26, 27]. In fact, Colombia has the world’s second largest displacement situation after the conflict in Syria. The forced displacement occurs due to extreme violence by armed groups such as guerrillas and paramilitary groups in the countryside. This violence includes the recruitment of minors, sexual violence, the deployment of anti-personnel mines, kidnapping, and massacres of entire villages [26, 28]. Both guerrillas and paramilitaries cultivated the trade of drugs as a means of obtaining resources for decades [28–30]. In spite of the peace agreement with FARC (Fuerzas Armadas Revolucionarias de Colombia, Colombian Revolutionary Armed Forces) guerrillas and a failed peace process with ELN (Ejército de Liberación Nacional, National Liberation Army) guerrilla group, this history is affecting and will affect Colombia for a long time, especially in the rural areas. In order to understand pregnancy in adolescence in this specific population of internally displaced women, it is necessary to understand how the context of armed conflict and displacement in Colombia affects the life of girls.

Despite extensive scholarship on adolescent pregnancy generally, there is little known about how internally displaced women experience and make sense of their own pregnancies during adolescence against a background of conflict and displacement [31].. Therefore, an ethnographic approach makes important contributions to the understanding of pregnancy in adolescence among IDPs and to inform and guide the development of policies and programs.

## Methods

To gain in-depth insight into the lifeworld of adolescent pregnancy from the perspective of displaced women in Colombia, we used an ethnographic approach. We used this approach because it is holistic, contextual, reflexive and presented from the emic perspectives, meaning from the perspectives of the members of the cultural groups involved (Trochim, 2006).

We performed in-depth interviews with a focus on life stories and participant observations. Alongside that, we organized workshops with the internally-displaced women (which were also open to other women from the receiving community) which enabled a process of sharing and exchanging experiences in a safe atmosphere, to capture their experiences, their perceptions of armed conflict, displacement and motherhood. We expected that these workshops would provide more in-depth insights than those we had gained from the individual interviews and would also confirm the findings from the interviews. Although the workshops were open to other women from the receiving community, only responses from internally-displaced women were analysed, in order to focus on their experiences of the displacement and its effect on their experiences of adolescent pregnancy.

### Setting and participants

Research was conducted in Ciudad Bolivar, Bogotá. Ciudad Bolivar is located in the southwestern part of the Capital District in the 19th locality. It has approximately 713,000 inhabitants. The area is about 13 million square meters, and 73% of the area is rural [32]. Its urban area is the one that receives the majority of IDPs in the capital. Of this population, 94% of the people fall into the lowest socioeconomic status. Ciudad Bolivar is known as one of the most violent parts of Bogotá, with a murder occurring every other day [33]. In this area, cells from illegal armed groups are operative [32–34].

Participants were internally displaced women, all living in the community, who were mothers during adolescence, before the displacement, but their age at the time of this study varied from 18 to 35 years. We chose the definition of adolescence according to the World Health Organization, that is “young people between the ages of 10 and 19 years” [7]. Most of these mothers had their first child at age of 14. At the time of data collection, they had between one and six children.

### Recruitment, data collection, data analysis

Research participants were invited by a local program known as Vidas Móviles. This program has been in the community for 12 years since 2006, and it is part of the service function of the Pontificia Universidad Javeriana. For a number of years before the study, the first author worked as a project physician in the community and she was known by some of the community people who were the key informants, which enabled her to invite participants via a snowball sampling strategy until saturation point. Fieldwork involved the first author visiting the community two or three times per week between June 2015 and May 2016, and irregularly (twice per month) in the following 2 years.

All participants were interviewed by the first researcher individually in Spanish, and all the interviews were recorded. Interviews were open, giving participants the freedom to tell their life stories, and were guided by the following five themes: original family, early pregnancies, armed conflict, displacement, and gender norms. These themes were inspired by several years of fieldwork in the community, as well as by relevant literature.

Furthermore, the first author organized eight workshops with 20 IDP women interviewed but was open to other women from the receiving community (however, only data from IDP women were included in the analysis). The workshops had 18 to 30 participants. Six of the workshops were dedicated to activities, such as painting, writing, writing on the wall, singing, and role-playing. The workshops facilitated communication among the mothers and allowed them to elaborate freely on their experiences and their ideas about topics that were discussed in the interviews. These dynamics permitted them to share their opinions freely and openly and also identify similar experiences of other participants. The first 6 workshops took place in the first year of field work, one every 2 months. The last two workshops were intended to have a feedback of the analysis of this study and took place in the first semester of the second year of fieldwork.

Most interviews were conducted in the houses of participants and others were done in the community house of the project Vidas Móviles. The workshops were also conducted in the community house except one which took place in the house of one participant.

Participants were displaced by different armed groups. During the workshops, the identities of the people who caused (violent) displacement were kept anonymous to facilitate the engagement of the participants. Some of the materials that were a result of the workshops were kept by the participants and others were stored in a safe place. Photographic records were taken of all the materials.

We used the thematic analysis proposed by Miles et al. (Miles & Huberman, 1994), that includes a repetitive and simultaneous process of reducing data (review and become familiar with the data and codification), displaying data (organizing and compressing assembly of information), and drawing and verifying conclusions (Ibid.).

To analyse the data, the life stories, interviews and workshop stories were transcribed in Spanish and entered into the Nvivo program to facilitate further data coding and analysis. After a first round of close reading by the first author, important themes and fragments were identified, discussed with the other authors, and refined. In order to receive feedback regarding the adequacy of the interpretations, the analysis was presented to and discussed with the community in two additional workshops. For the publications, relevant quotes were translated from Spanish to English.

### Ethical framing

Research and ethical approval was granted by the Ethic Committee of the School of Medicine of the Pontificia Universidad Javeriana, Bogotá (Act 21/2015.FM-CIE-8744-15). In the same way, research protocol and methods were consistent with the Colombian Law. All participants had their first baby during adolescence, but at the moment of the interview their age was from 18 to 35 years old. Participants were asked to provide and to sign informed consent. We took care that the participants were fully informed about all aspects of the project and were aware that they could withdraw at any moment without providing reasons, and that eventual withdrawal would not affect their relationship with the community project Vidas Móviles in any way. Recordings were destroyed, and transcripts will be stored in accordance with Dutch Law in a protected location at Maastricht University for 10 years.

This research followed the ethical recommendations from the American Anthropology Association and continuously reflected on possible ethical concerns during the study that go beyond informed consent. We protected the identity of the participants by changing their names in the text. In addition, we did not identify the violent groups that are mentioned as causing displacement. We took care of the participants by following up during monthly workshops and helping them with some difficulties they were facing (e.g., helping them to access the aid offered by the government, finding schools for their children, or attending to certain questions related to health).

## Results

In this section, we will analyse the four main themes that came to the fore in the life stories of the participants as well as in the workshops, namely experiences of rural violence during their early lives, family life during their childhood, stories about becoming pregnant at an early age, and experiences of responses of family members and partners. After presenting how the women look back and give meaning to their stories about pregnancy in adolescence and their lives as young mothers in retrospect, we will discuss how our findings provide insight into the ‘possible pathways of pregnancy’ and relate them to relevant literature.

*1.* *Rural violence:* ***‘They (the armed groups) are the owners of the village. There is no other law or rule … you cannot imagine what I’ve been through….’***

According to the National Planning Department in Colombia [34], the rural areas in Colombia have worse life conditions in comparison to the cities. This is not only because of the armed conflict, but also because of the poverty and lack of availability of health and education in those areas.

When invited to share their life story all interviewees, without exception, point to the violence in the villages and the history of later displacement as major experiences. The women narrate an atmosphere of violence in the rural areas and some shared experiences of extreme violence and massacres due to the armed conflict. Participants explained that the guerrilla or paramilitary groups had control over the villages and the population. Farmers were forced to give these groups food and resources, women suffered sexual assault by these members, and young people were forced to be part of the groups. Some of the women explained how these groups took family members when they were children and how they asked for support using violence, threats or by killing people.

*“We started to build our house. We worked on a farm with coffee, yuccas, and plantains … but they (armed groups) suddenly knew that we were there, so every week they came asking for food, cassavas, or two or three banana bunches. They took our maize, and they left our farm destroyed … Once I was at home, washing clothes, when they arrived. My husband was in the field, and one of the men wanted to have sex with me … my daughter was sleeping … so I started to fight with them. I told them that our house was not their house and that they should leave us … The man took me, but at that moment, my husband arrived, and he became really angry with the guy and tried to hit him … But the other guy took out a gun and put it to my husband’s head … At that moment, one of the head men of the group arrived … fortunately, he stopped the other men … and they left. But at 8 p.m. … they were coming … They almost killed my husband … he said, ‘pack some clothes, we will go.’ … So Peter told me that he will call his father to bring a horse to carry us and our clothes. His father arrived at 11. We packed some clothes for our girl and something for us … we arrived at 3 a.m. My mother-in-law cried. That day, Peter got some money for the bus, and we came here (Bogotá), that day”.* (Luisa, in-depth interview)

For some women, violence - rape - was the cause of their pregnancy. The stories of the women showed how they and their family members more or less inevitably got involved with armed groups and violence because they lived immersed in the violent environment that represents the armed conflict in Colombia.

*2*. ***Early family life: ‘I think my pregnancies are because of that … all the things I lived through at home.’***

Next to the violent atmosphere in their communities due to the armed conflict, all interviewees tell that their family circumstances during childhood life were difficult from the start. They not only recount neglect, suffering, and a lack of care in their early family life, but also violence and rape. The women see these family circumstances as important determinants of their pregnancies. The idea that pregnancy in adolescence relates to this kind of suffering during childhood was shared by all participants.

The story of Betty, a 23-year-old woman, is illustrative of this belief:

*The relationship with my mother was really bad. She always mistreated me. She told me that I am not useful for anything … She hit me all the time … with anything she found, cable, sticks, or even a machete … She liked to put me on the floor, with her foot on my neck, and hit me very hard … She didn’t hit my brothers … only me. When my father died, my mum sent me to my Aunt Ursula, but she hated me. Ursula wanted to kill me … I was 9 … she hung me off the rafters and hit me with everything … she left me and her younger children locked in the room, without food, extremely hungry. And you know … we were children … we saw the neighbour that prepared a lot of food. Sometimes they threw the food away... and I started to steal the food … sometimes we escaped to look for food in the garbage. My aunt arrived usually at 5 or 6, and we were locked in the room … I can’t forget those years … they were very sad... also when I went to live with my Aunt Ursula, her oldest son, with a cousin, tried to rape me too. So it was very hard for me. I didn’t want endure that, all that mistreatment, the hits, and all the abuse … so I made the hardest decision I ever had made. I left home.* (Betty, in-depth interview)

It is striking that while one would expect that parents, family members, and caregivers would be protective and helpful in cases of pregnancy in adolescence, the opposite is true. The characteristics of family life contribute to a situation in which teenage girls become vulnerable to pregnancy in adolescence. While rape appears to be a common experience for the participants, they also mentioned a lack of support from others when the rape happened. This is the case of Juana:

*“I was 13 years old. That man (my stepfather) was terrible. He hit us, mistreated us … every night he arrived drunk to abuse us. It was very difficult. He called us bitches and all kinds of bad words. We usually went to bed with our clothes on … with shoes on. We were ready, waiting for him … I told my mum about the rape … but she didn’t trust me. She said that it was my fault, that I wanted to be with him … and I answered her that if I wanted to be with him, I wouldn’t have these wounds and all the pain that I have … and we said to my mum, ‘we don’t want to live with him,’ but she said to us that he will change … and then, that week, he arrived drunk again … ‘please don’t hit my mum’ … so my sister took a knife to defend my mum, and then the man was looking for my sister to kill her … we were forced to leave our home in the middle of the night …’* (Juana, in-depth interview)

Most participants in our study explained that they got involved in early relationships with men (even from different armed groups) as a way to escape from indifferent, hostile, or violent circumstances at home. They told that entering into relationships with men at early age was at that time seen as a way to avoid the mistreatment received at home. In their stories, women constructed pregnancy in adolescence as a result of the violence experienced during childhood and the need to flee from home.

*‘I was living with my mum, my uncle, and my brother … but I had a “dog’s life” (mistreatment) … I had to do the cleaning … and if something was wrong with the cleaning, I didn’t receive food … He left me sleeping on the floor, very cold … I made the decision to leave my home at 11 years old … I thought it was a good decision, but it was the worst thing I could do … I left my home to go with a man much older than me … I was 11, and I was rebel. I think all of this led me to be a mother so early. I didn’t think … about the bad life, the needs, the abuses … but then I didn’t know that my partner was an addict. He was much older than me’*. (Silvia, in-depth interview)

The life stories of the interviewees tell us how some of the women perceived their childhood as an important and determinant experience that, on many occasions, led them to leave their homes while they were still very young. This linked with ideas of ‘genuine love affair’ where women trust men, are the main explanation to get pregnant in an early age, from participants point of view, as was explain by Villaran, 2017 [5]. This is what happen to our participants, where this decision to escape from home and engage into a relationship lead them to get pregnant.

In addition, many of participants had different children with different partners. It could be explained by the desire they had to build a family and experience stability, as some authors have argued [30]. This is the case of Sandra who explained as following:

*‘I was 17 when I got pregnant again. The father of my first baby abandon us, so I returned to my mother and stepfather, and I started to work in cleaning … and then I meet the father of my second child. He worked in a coca crop. He told me that we could live together and that he would take care of my first child. I trust him. I got pregnant of him, and when I told him, he said that he already had another woman.’* (Sandra, in-depth interview).

*3* ***Reactions to pregnancies: ‘When my stepfather noticed that I was pregnant, he slapped my face and told me, ‘You are a whore and that is why you are pregnant.’***

In narrating their life stories, the girls expressed that they did not know that they were pregnant for quite a while and that the pregnancy was unexpected. In general, most of them were only at the age of 14 or 15; they expressed that they felt panic and distress and needed understanding and help. However, in line with the stories about a lack of care during early childhood, women felt that they did not receive support from their original families; rather, they received negative and punitive reactions from their parents and siblings regarding their pregnancies.

*“I was 13 and pregnant, but I didn’t know … I had sex with a boy, and I got pregnant, but I was not a virgin because my stepbrother had raped me, but I never told anyone about that. So … after two months, my breasts started to itch … and sometimes I had liquid come out of a breast … so my mum shouted at me … ‘Don’t tell me that! You are pregnant’ and she grabbed my face. And later she threw me out of the house … I pleaded with her … ‘Please don’t do that. I can do whatever you want,’ but she said, ‘no,’ because here in Bogotá, people don’t rent rooms with children, and you knew that it is bad … My mum kicked me out of the house … she said to me ‘go look for a house of a friend because you opened the legs, so you must work to deal with that”.* (Monica, in-depth interview)

When we inquired about family planning methods, some mothers said that they did not pay attention about family planning before their pregnancies and also expressed that in the countryside, family planning is not an important topic. Nevertheless, other women expressed that they became pregnant even if they had some knowledge about how to prevent pregnancy because they wanted to please their partners.

*‘Yes, I knew about condoms and those things … but in the village, you don’t mind so much to that … for example; you put the condoms full of water in your room to protect you from mosquitos (laughing) … .’* (Olga, in-depth interview)

*‘You know, when they (men) ask for a baby, you must to give it to them … otherwise, they will ask it from another woman.’* (Sonia, workshop)

Some participants expressed that talked to their mothers about sex or family planning methods were difficult, because is still a taboo. For example, Sandy said:

‘*My mom never told me about sex or ay method. You should not date with boys, and that is it. Those are topics that you learn at school.*’ (Sandy, in-depth interview).

While family members were not that positive when they got pregnant, the fathers of the babies (i.e., the men or boys that had relations with the girls) often were not very helpful either. Most stories displayed a similar pattern. Women described that they often entered into relationships to escape violence in their homes or in their villages, searching for shelter, safety and happiness. Their partners were farmers or even members of the armed groups. The women explained that when they became involved in relationships, the men were enthusiastic, friendly, and caring. However, after some time, especially when the women became pregnant, the men changed. Women were abused and mistreated, and their partners were unfaithful to them or even abandoned them.

*My husband left us 7 months ago … with another woman … they started at parties, dancing and drinking … and I have suffered a lot because … you know … he has been my only man, and I have cried a lot. I went to his mum’s place and asked him to come back with us … I begged him ‘don’t leave us,’ but he refused. So, I decided to go …* (Patty, in-depth interview)

During the workshops, looking back to their lives, many of the participants explained that the role of a Colombian woman is more difficult than the role of a man because a woman needs to work, take care of the children and the house, and please the man. Men only work and want to be pleased by women, and have children. Regarding this “Macho” society, as alluded to by Quevedo [35], men are socially allowed to have other women. In the case of our participants, sometimes they knew about their partner’s unfaithfulness, but they decided to stay with their partners due to economic support.

*‘I think the men just wanted to abuse you … It is very hard, but you know … sometimes you accept because you don’t have any money.’* (Sandra, workshop)

*‘Yes, after my little boy, I got pregnant again. I realized that when I was 3 months pregnant. But at that moment, I knew that my husband had another woman, so I talked with him. He asked me for forgiveness, and I forgave him … because you know … what you can do with the children alone?’ (*Laura, in-depth interview)

*‘That is true … I think as a woman, your life is difficult. For the men, life is easy … and it could be worse if you are a single mother.’* (Paola, workshop)

From the narratives of the women, it was clear that their partners did not take care of them and their children. When partners left them, it could be for different reasons: their partners were in jail, were killed, or just left because of another woman. Some of the women explained that they as women have had different partners during their lives, and they were sometimes looking for support for their children. The oldest participants had five to six children and, to a fairly large extent, had to take care of their children on their own.

*4* ***Meanings of pregnancy: ‘That was the best experience of my life … but not really.’***

In the workshops we invited the interviewees to explain what pregnancy and motherhood at a very early age meant to them. Surprisingly, considering the life stories of the women, some of the women described motherhood as a beautiful experience that offers the opportunity to give love and care for children. Motherhood for them represents a chance to build a family.

*‘Being a mother is a beautiful experience … you know … to have a baby, something that is yours and you can take care of … the only thing is that you need to work hard to feed them and try to give them all the things you didn’t receive.’* (Lourdes, workshop)

They also expressed that they would like to meet all of their children’s needs and prevent them from suffering. However, they also reflected in their life stories that they were sometimes frustrated or dissatisfied by how they were raising their children. Women noted that their children felt they lacked their mother’s time, attention and love; that they experienced deprivation; and that they, too, were affected by the displacement.

*‘My children are rebellious because of everything … my son is an addict … My daughter is now a young mother, as I was, and she has no stability. I couldn’t do anything for them. I knew that they needed me, but they also shouted at me that they hated me … I once thought of suicide … I think people cannot understand all the things that happened to me, the needs, abuses, rapes … I think my children are like this because of the displacement.’* (Francisca, in-depth interview)

From the workshops, we found that the ideal of a nuclear and happy family is not always a reality. Nevertheless, as young mothers, participants wanted to give their children everything they can so that they would grow up as “good persons.” Participants also shared the opinion that being a mother represents a challenge that you cannot escape but gives to you a reason to continue in life.

## Discussion

In this article we analyse the experiences of displaced women with adolescent pregnancy in Colombia and explore the ‘pathway of pregnancies’ they had from their point of view. Internally displaced women explained their ‘pathways of pregnancies’ as being shaped by the difficult rural context in an environment of armed conflict where they were born and raised. Just as importantly, they highlighted the role of violent family circumstances during childhood that could be interconnected. Despite this, their ideals of having a beautiful family and putting trust in another opportunity to rebuild their lives guided them to escape from home and get into relationships that resulted in early pregnancies, whether desired or not. To participants, motherhood represented an ideal opportunity to achieve fulfilment; nevertheless, breakdowns led them to challenges in being ‘good’ mothers and supplying all the needs of the children.

Notably, rural violence was a major theme of participants’ life stories. Being in an armed conflict context represented traumatic experiences for participants. They were forced to satisfy the needs of members of armed groups, including providing sexual favours, in order to survive. At the same time, sexual violence was used as weapon of war. It is in the same line as some scholars as Ortiz, Wilches and Alzate, who have identified these patterns [28, 36, 37]. Participants also cited structural and interpersonal violence within home settings. Structural violence in the form of poverty and lack of opportunities is well-documented in Colombia and is worse in rural than in urban areas [34]. Childhood experiences of poverty, lack of opportunity and interpersonal violence have been reported by women who experienced adolescent pregnancy in contexts as varied as the Democratic Republic of Congo [38]. Authors such as Zanchi [39] and Harden [40] suggest that girls living in unfavourable contexts, (i.e., who obtained little education, had limited future employment prospects, or lived in otherwise difficult circumstances), were likely to live in similar situations as adults and often understood early entry into motherhood not as a problem, but as a natural progression in life that would not cause them harm. This sentiment was echoed by participants in our study.

In addition, women reported that experiences of interpersonal violence at home often pushed them to leave their family home, and to seek romantic relationship with a male partner (who may have been a member of an armed group) in order to experience safety and stability. Many of the participants in this study reported early experiences of sexual coercion and violence. Interpersonal and sexual violence is also widely reported in the literature as a contributing factor to early pregnancy [41–43]. Going back to Kelly’s typology, this study could be part of type A and B, because of the traumas lived by participants, and also because of the history of violence residing in the families of origin [21].

After leaving their homes, women described trying to build a family and getting into relationships with men, but noted that they often fell into abusive relationships, trying to escape again and repeating the story. That is why, they explain, they have many children with different fathers. Similar to our findings, some authors have explained this situation of desire for love or normalcy of family in different settings [44–46]. From the participants’ stories, it become clear that the lack of support of the fathers of the babies was significant. This is consistent with findings from other research about early pregnancy [20].

Furthermore, our participants shared how on becoming pregnant, they lacked support and were on their own. Their stories showed how they need social support in order to face the challenges of pregnancy and motherhood. Similarly to our findings, some authors have pointed out how young mothers felt being neglected by parents and other adults or how they lack social support [44, 47, 48].

Regarding their perception of teenage motherhood, participants expressed ambiguities based on their experiences. On the one hand, pregnancy was an opportunity to build a family and to experience love in an often hostile environment. On the other hand, it was extremely difficult to raise children alone. These ambiguities are shared with women in different settings, where interview respondents indicate that motherhood helped girls to be better persons and increased sense of responsibility [49, 50]. Other young mothers have described adolescent pregnancy as a positive life event that transitioned them into adulthood and provided opportunity for personal growth [51–53]. This is comparable to our findings. Going back to Kelly’s typology, participants’ stories conveyed the desire to build a family alongside notions that becoming a mother can lead them to an adult status, leading us into type C [21].

### Limitations

As a qualitative approach, the empirical findings cannot be generalized to other populations in other contexts. We analysed life-stories, which are the construction about their lives, from their perspective in a moment of life. It is specifically on how they constructed on their own lives. In that sense, there is not recall bias, because it is not about the “true”, but instead, it is about on how they construct their lives, even if the pregnancy or the displacement occurred long time ago.

Coding was only conducted by one person, nevertheless, the emerging themes were discussed with other authors. Similarly, eventual bias is also reduced because all data and interpretations were discussed with the other members of the research team, who were not part of Vidas Móviles project.

People from the neighbourhood know the first author as a physician and as part of the project Vidas Móviles. Participants in the study expressed that condition as a plus, because they trusted the researcher and felt that they could share their experiences, including medical issues.

## Conclusion

This study demonstrates the ambiguities and complexities in which pregnancy during adolescence among internally displaced women occurs, from their perspectives. Participants characterized their early pregnancies as being inextricably tied to not only the violence suffered as a result of the armed conflict in the rural areas, but primarily to the violence they suffered within their original families. According to the analysis, early pregnancies brought them stigmatization but also feelings of challenge and ownership. Pathways of pregnancies among internally displaced women are complex and go beyond mainstream discussions of adolescent pregnancy, which primarily focus on contraception methods and/or family planning strategies. This study provides insights of realities of this specific population and their perspectives regarding their experiences as young mothers.

## Data Availability

Transcripts will be stored in accordance with Dutch law in a protected location at Maastricht University for 10 years. The datasets generated and/or analyzed during the current study are not publicly available due to the sensitive nature of the topics in a population who was part of the armed conflict in Colombia, participants were assured raw data would remain confidential but are available from the corresponding author on reasonable request.

## Abbreviations

ELN: Ejército de Liberación Nacional (National Liberation Army)
FARC: Fuerzas Armadas Revolucionarias de Colombia (Colombian Revolutionary Armed Forces)
IDPs: Internally Displaced Persons
